# Production of aqueous spherical gold nanoparticles using conventional ultrasonic bath

**DOI:** 10.1186/1556-276X-7-420

**Published:** 2012-07-27

**Authors:** Ji-Hwan Lee, Stephen U S Choi, Seok Pil Jang, Seoung Youn Lee

**Affiliations:** 1 Department of Mechanical and Industrial Engineering, University of Illinois at Chicago, Chicago, IL, 60607, USA; 2 School of Aerospace and Mechanical Engineering, Korea Aerospace University, Goyang, Gyeonggi-do, 412-791, Republic of Korea

**Keywords:** Spherical gold nanoparticle, Ultrasonic bath, Ultrasonic energy

## Abstract

A conventional ultrasonic bath was used to examine the feasibility of forming aqueous spherical gold nanoparticles (GNPs) under atmospheric conditions. The effects of ultrasonic energy on the size and morphology of GNPs were also investigated. Highly monodispersed spherical GNPs were successfully synthesised by sodium citrate reduction in a conventional ultrasonic bath, without an additional heater or magnetic stirrer, as evidenced by ultraviolet–visible spectra and transmission electron microscopy. Ultrasonic energy was shown to be a key parameter for producing spherical GNPs of tunable sizes (20 to 50 nm). A proposed scheme for understanding the role of ultrasonic energy in the formation and growth of GNPs was discussed. The simple single-step method using just a conventional ultrasonic bath as demonstrated in this study offers new opportunities in the production of aqueous suspensions of monodispersed spherical GNPs.

## Background

Gold nanoparticles (GNPs) have generated much interest due to their unique and attractive physical and chemical properties, such as high thermal and electrical conductivity, photothermal effects, tunable size and shape dependent optical properties, chemical stability, biocompatibility and facile functionalisation, and are used in a wide range of applications including material science, catalysis, biomedicine, and quantum dots technology [1-6]. Since the first scientific research on the formation of gold colloids by the reduction of gold trichloride by phosphorus was published by Faraday in 1857 [7], various methods for the synthesis of colloidal gold have been used, such as chemical methods [8-11]. The well-known Turkevich method [8,12] is the simplest way to produce aqueous suspensions of monodispersed GNPs with good stability [13].

Sonochemistry has also been used to synthesise colloidal gold since the pioneering work on the formation of GNPs using ultrasonic sound was carried out in 1980 [14]. Extensive studies on the sonochemical production of GNPs have been performed to investigate the effects of many synthesis variables on the size of GNPs [14-21]. These studies show that most GNPs have been synthesised in non-aqueous solutions using a high intensity ultrasonic generator. For example, various alcohols were used as the base fluid, reducing agent, and stabiliser in the greater part of sonochemical works [14-18,20]. However, with regard to GNPs synthesised in aqueous solutions, there has only been limited research. It has been reported that the rates of the formation of GNPs in pure water were approximately zero without any additives such as surfactants, water-soluble polymers and aliphatic alcohols and ketones under atmospheric conditions, resulting in only a small amount of synthesised GNPs that were unstable and coagulated within several hours [16]. Horn or cup-horn type ultrasonic generators were used in previous studies [14-20] to apply sufficient ultrasound energy to cause the pyrolysis of fluid molecules. An appropriate ultrasonic energy is required to induce collapsing gas bubbles with high temperature (in excess of 4,000 K) [22,23]. It also has been reported that the reduction of gold (III) occurred when using a high intensity ultrasonic generator, but this did not occur when using a conventional ultrasonic bath [17].

The present study is interested in producing GNPs in water, not in an organic medium, because GNPs used in biological applications are in water. Hence, it is of practical interest to synthesise aqueous GNPs in a simpler but more consistent process using a conventional ultrasonic bath instead of a horn or cup-horn type ultrasonic apparatus. The importance of the synthesis of aqueous GNPs is well-summarised by Ji et al. [24] as follows: (1) High-quality gold nanocrystals have been synthesised in non-aqueous solutions under elevated temperatures; (2) however, from a green chemistry standpoint, all non-aqueous synthetic schemes are far from ideal; (3) water may eventually become a plausible medium for the growth of high-quality nanocrystals with various compositions [25]; and (4) this attractive future will likely come with systematic and quantitative studies of some carefully chosen aqueous model systems such as aqueous gold nanocrystals synthesised by citrate reduction. In addition, a conventional ultrasonic bath may become a simple apparatus for the production of consistent quality spherical GNPs in aqueous solutions. However, Nagata et al. [16] and Okitsu et al. [17] have shown that it is barely possible to synthesise stable GNPs in pure water using a conventional ultrasonic bath under atmospheric conditions.

Recently, Chen and Wen [26] proposed a novel ultrasonic-aided method for the synthesis of aqueous gold nanofluids containing both spherical and plate-shaped GNPs and demonstrated that their shape and size were controllable. They synthesised aqueous gold nanofluids containing spherical GNPs by the conventional citrate reduction method, and then placed the gold nanofluids in an ultrasonic bath to study the effect of sonication time on nanoparticle size. They also synthesised aqueous gold nanofluids containing plate-shaped GNPs by citrate reduction of chloroauric acid (HAuCl_4_) solutions immersed in an ultrasonic bath at room temperature to study the effect of sonication time on the morphology of the gold materials produced.

In this work, only a conventional ultrasonic bath was used without an additional heater or magnetic stirrer under atmospheric conditions to examine the feasibility of forming aqueous GNPs by sodium citrate reduction. Although ultrasonication was used in this study in Chen and Wen [26], its effect is quite different. Since we produced spherical-shaped GNPs in the presence of ultrasonication, we were able to see the effects of sonication on the formation of spherical-shaped GNPs. In contrast, such effects were essentially non-existent in the investigation of Chen and Wen [26] because they sonicated aqueous spherical-shaped GNPs that had been formed and grown in the absence of ultrasonication. We also examined the effects of ultrasonic energy on the size and morphology of GNPs and discussed a proposed scheme for understanding the role of ultrasonic energy in the formation and growth of spherical GNPs in an ultrasonic bath. The present study shows for the first time that aqueous spherical GNPs can be produced by sodium citrate reduction in a conventional ultrasonic bath without any additional heater or magnetic stirrer. This single-step synthesis of aqueous GNPs using a conventional ultrasonic bath allows us to investigate the effects of sonication time and ultrasonic energy on the formation and growth of GNPs.

## Methods

### Production of gold nanofluids using an ultrasonic bath

**Figure 1:**
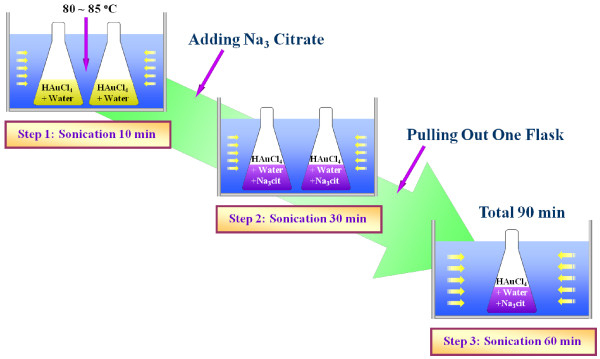
Schematic of aqueous gold nanoparticle synthesis procedure by modified citrate reduction method.

Two separate but identical aqueous HAuCl_4_ solutions were prepared by adding HAuCl_4_ (Sigma-Aldrich Corporation, St. Louis, MO, USA) to two conical flasks filled with 200 ml distilled water (J.T. Baker Chemical Company, Phillipsburg, NJ, USA). The HAuCl_4_ concentration was set to 0.25 mM [24,27,28]. A conventional ultrasonic bath (VWR Aquasonic 150 T, 40 kHz, 135 W, VWR International, LLC, Radnor, PA, USA) was filled with 4.5 L of water at 80 °C. Due to ultrasonication the temperature of the water was slightly raised and maintained at 80 °C to 85 °C during the synthesis of the GNPs in order to produce GNPs of the smallest possible size using the citrate reduction method [8]. The two flasks containing the identical aqueous HAuCl_4_ solutions were immersed in the ultrasonic bath for 10 min to stir and heat up the solutions. The flasks were capped during sonication to prevent the loss of solution due to evaporation. After 10 min sonication time, sodium citrate (C_6_H_5_Na_3_O_7_) was added to the two aqueous HAuCl_4_ solutions. The molar ratio between HAuCl_4_ and C_6_H_5_Na_3_O_7_ was set as 1:3.5 to synthesise the smallest GNPs [24]. The molar ratio between HAuCl_4_ and sodium citrate is an important factor that affects the size and morphology of synthesised GNPs. The effects of molar ratio between HAuCl_4_ and sodium citrate on the size and morphology of GNPs are well presented in [12,24]. The traditional molar ratio between HAuCl_4_ and sodium citrate to produce approximately 20 nm-sized GNPs with the conventional citrate reduction (CR) method is almost 1:3.5 as stated in [12,24], and many research groups have successfully reproduced approximately 20 nm-sized GNPs with the conventional CR method at almost 1:3.5 ratio [12,24,27]. To investigate the effects of sonication energy on the size of gold particles produced, one of the mixtures was sonicated for 30 min after adding C_6_H_5_Na_3_O_7_, and the other mixture was sonicated for an additional 60 min, as shown in Figure 1.

Our method to produce spherical-shaped GNPs is unique, mainly because the starting materials we used and the shape of GNPs we produced are quite different from those described in the experimental study of Chen and Wen [26]. First, the starting materials to which we applied sonication to make spherical-shaped GNPs were chloroauric acid, sodium citrate and water. In contrast, the starting materials to which Chen and Wen [26] applied sonication were aqueous 20 nm spherical-shaped GNPs that were already synthesised in the absence of ultrasonication by the conventional CR method. Therefore, it can be inferred that ultrasonication did not affect either the formation or growth of spherical-shaped GNPs. Second, when ultrasonication was used from the initial CR stage, the results obtained by our method and that used by Chen and Wen [26] are different. The GNPs produced by the other method were plate-shaped rather than spherical-shaped. In contrast, we produced spherical-shaped GNPs. It should be noted that the primary aim of our work was to synthesise aqueous spherical GNPs in a conventional ultrasonic bath. The difference in the shape of GNPs was due to the different reaction temperatures and molar ratios of HAuCl_4_ to sodium citrate as follows: (1) We maintained the reaction temperature at 80 °C to 85 °C during the entire process of synthesis of GNPs in our study, while Chen and Wen [26] performed experiments at 25 °C and exposed the resultant solutions to natural light for 16 h further; and (2) we set the molar ratio between HAuCl_4_ and sodium citrate at 1:3.5 based on the study by Ji et al. [24]; while Chen and Wen [26] used a different molar ratio based on the study by Huang et al. [28]. As a consequence, we produced spherical-shaped GNPs, while Chen and Wen [26] produced plate-shaped GNPs. Since the focus of our study was on the spherical-shaped GNPs, we did not use their data on plate-shaped GNPs, although they are interesting and important.

Our method has two primary advantages over that of Chen and Wen [26]. First, our results presented in the next section show that ultrasonication alone is very effective in the synthesis of spherical GNPs. In this way, a heater/stirrer is not necessary to initiate nanoparticle formation and growth. Thus, we have developed a simplified method to produce size-tunable spherical GNPs. Second, because this simplified method involves fewer steps compared to the procedure used by Chen and Wen [26], it is a highly reproducible method for making consistent quality spherical GNPs.

## Results and discussion

**Figure 2:**
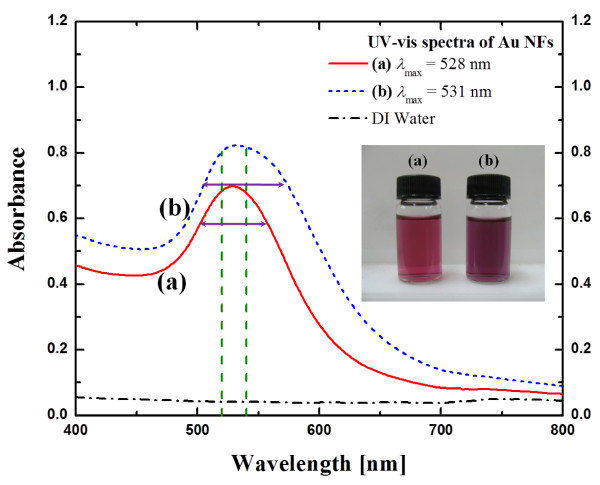
**UV-visible spectroscopy of synthesised gold suspensions.** (**a**) gold suspension (maximum absorption peak *λ*_max_ is 528 nm) prepared with 243 kJ (30 min with 135 W ultrasonic power) of sonication; (**b**) gold suspension (maximum absorption peak *λ*_max_ is 531 nm) prepared with 729 kJ (90 min with 135 W ultrasonic power) of sonication.

The final colours of the suspensions produced were reddish-violet or dark purple as shown in Figure 2. The two suspensions were capped and cooled naturally at room temperature. They still remained stable with little or no agglomeration after 2 months of storage. As shown in Figure 2 the ultraviolet (UV)-visible absorption spectrum of the two final products demonstrated the existence of spherical shape GNPs because the absorption peak was centred between 520 and 540 nm [24]. The plasmon band of 243 kJ (30 min with 135 W ultrasonic power) sonicated GNP solution is narrower than the 729 kJ (90 min with 135 W ultrasonic power) sonicated one. This implies that the size distribution of the GNPs of 30 min sonicated solution is more uniform than 90 min sonicated solution [21].

**Figure 3:**
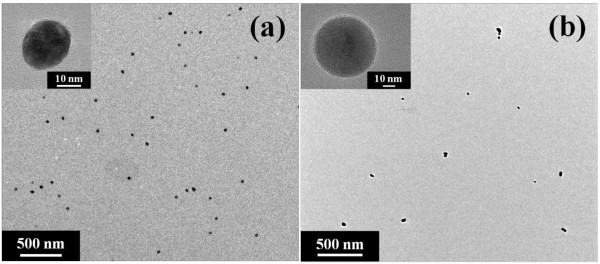
**TEM images of synthesised gold nanoparticles.** (**a**) gold nanoparticles (approximately20 nm) prepared with 243 kJ (30 min with 135 W ultrasonic power) of sonication; (**b**) gold nanoparticles (20 to 50 nm) prepared with 729 kJ (90 min with 135 W ultrasonic power) of sonication. Initial HAuCl_4_ concentration was 0.25 mM and molar ratio between HAuCl_4_ and sodium citrate was set at 1:3.5.

Transmission electron microscopy (TEM) images of these GNPs are shown in Figure 3. The TEM samples were prepared by Formvar stabilised with carbon-coated copper TEM grids and dried in air for 12 h after 2 months of gold nanofluid synthesis. The particle size of 243 kJ (30 min with 135 W ultrasonic power) sonicated solution was roughly 20 nm (21.7 ± 2.5 nm, average diameter ± standard deviation) and the size distribution was highly monodispersed, while the particle size and distribution of the 729 kJ (90 min with 135 W ultrasonic power) sonicated solution were broad (20 to 50 nm, 37.2 ± 12.4 nm) and polydispersed, as measured by TEM (JEOL JEM-2010 and JEM-2100 F, JEOL Ltd., Tokyo, Japan).

**Figure 4:**
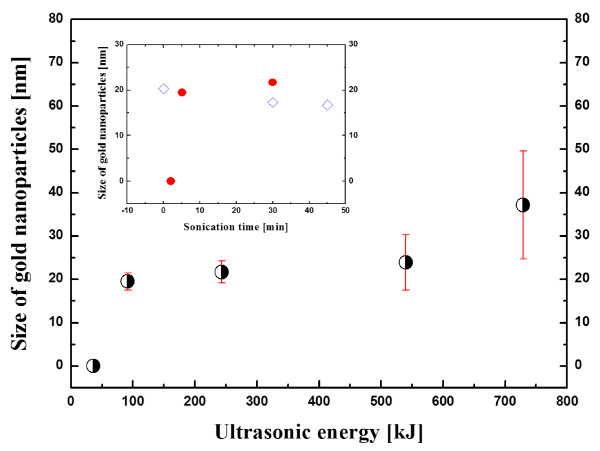
Average sizes of gold nanoparticles as a function of ultrasonic energy.

Figure 4 shows the effect of ultrasonic energy (sonication time × ultrasonic power) on the size of GNPs when using a conventional ultrasonic bath with fixed ultrasonic power and frequency (135 W and 300 W, 40 kHz). In Figure 4, we can see that the size of nanoparticles is critically dependent on the ultrasonic energy or sonication time at a given power. GNPs were not formed when the two aqueous HAuCl_4_ solutions were initially sonicated with 36 kJ of ultrasonic energy from the bath-type ultrasonic generator. However, spherical GNPs with a diameter of 20 nm (19.6 ± 1.0 nm, average diameter ± standard deviation) were successfully synthesised when the ultrasonic energy was increased to 91.5 kJ. The nanoparticle size was observed to slowly grow with increasing ultrasonic energy up to 270 kJ, and the size distribution is highly monodispersed. The nanoparticle size rapidly grows to nearly 50 nm (37.2 ± 12.4 nm) with higher energy beyond 540 kJ, and polydispersity also increases with increasing ultrasonic energy. As discussed in the next paragraph, particle-particle fusion is one possible reason for the size increase with increasing sonication time. The error bars in Figure 4 correspond to the standard deviation of the average size of GNPs. Since Chen and Wen [26] presented their data as a function of sonication time from 0 to 45 min, their data (hollow blue diamonds) are reproduced in the inset for comparison with the results of the present study (solid red circles). As shown in the inset, Chen and Wen [26] produced spherical GNPs with diameters of approximately 20 nm. Their results seem to be consistent with the results obtained in this study. However, we see an interesting difference in the size of nanoparticles during the first 2 min of sonication time due to the different synthesis procedures. As mentioned in the introduction, Chen and Wen [26] studied the effect of sonication time on nanoparticle size using aqueous gold nanofluids that were first synthesised by the conventional citrate reduction method and then placed in an ultrasonic bath. Therefore, the size of their GNPs was 20 nm at the start (0 min) of sonication. In contrast, in the present study because we sonicated the aqueous HAuCl_4_ solutions that were immersed in the ultrasonic bath, we were not able to find aqueous spherical GNPs of 20 nm synthesised at 2 min of sonication time.

**Figure 5:**
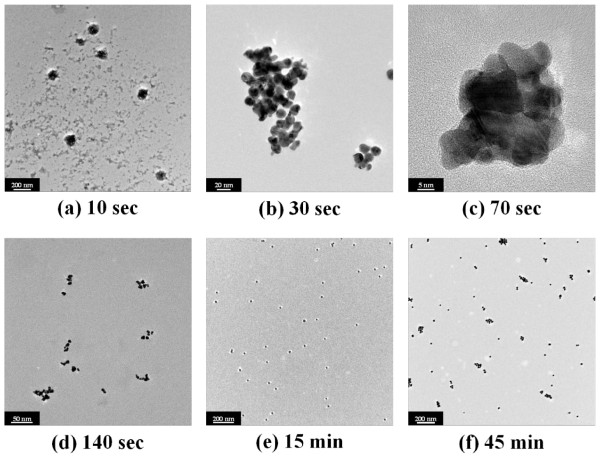
**TEM images of gold nanoparticles formed by citrate reduction as function of elapsed time.** TEM images of gold nanoparticles formed by citrate reduction as function of elapsed time when using a conventional ultrasonic bath (40 kHz, 300 W): (**a**) after 10 sec, (**b**) after 30 sec, (**c**) after 70 sec, (**d**) after 140 sec, (**e**) after 15 min; and (**f**) after 45 min. Initial HAuCl_4_ concentration was 0.25 mM, and molar ratio between HAuCl_4_ and sodium citrate was set at 1:3.5.

**Figure 6:**
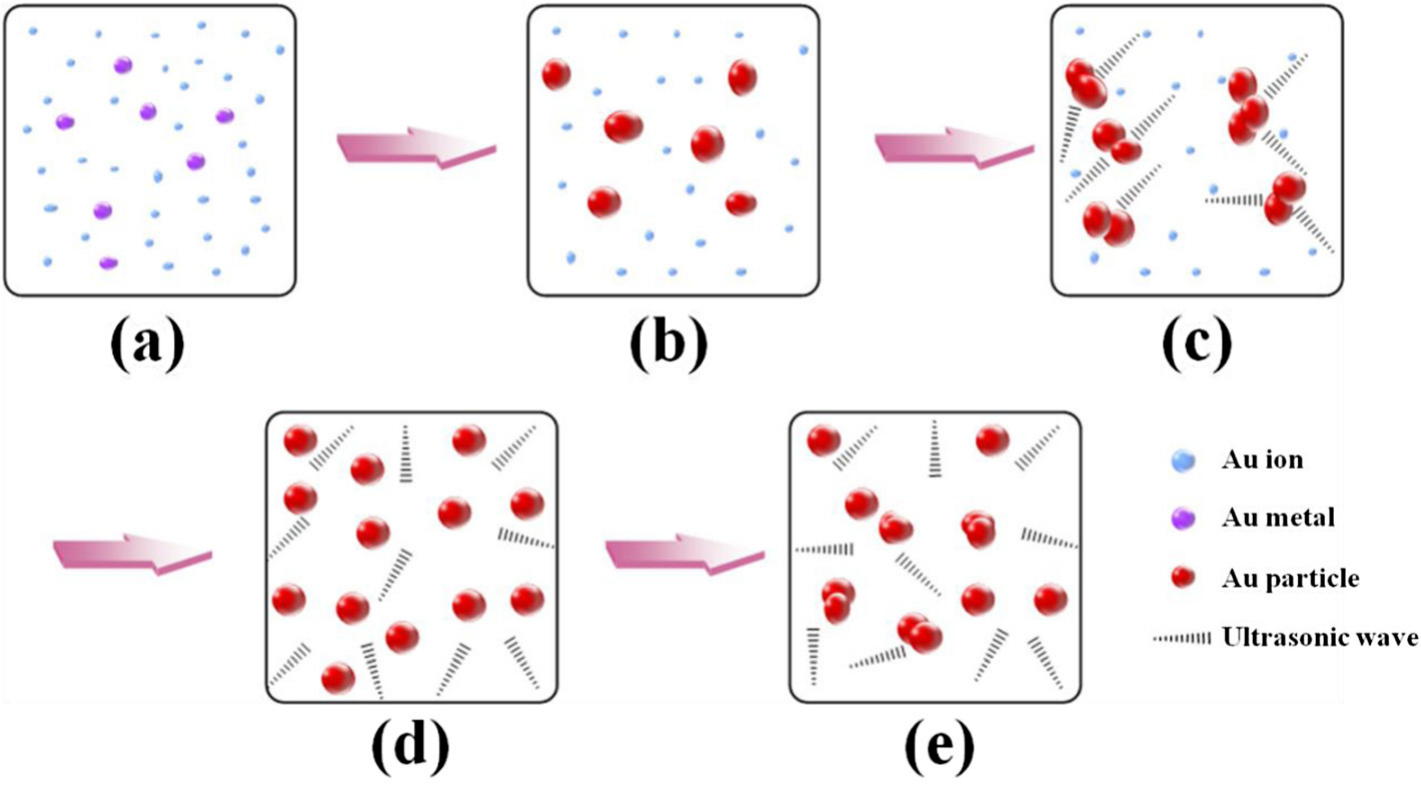
**Proposed scheme for understanding the role of ultrasonic energy in the formation of gold nanoparticles.** (**a**) Reduction of gold ions with sonication energy;, (**b**) growth of gold nanoparticles, (**c**) prevention or minimisation of wire-shaped gold cluster formation by breaking chain-like nanowires by ultrasonic waves, (**d**) monodispersed gold nanoparticles by ultrasonic waves with Oswald ripening or intra-particle ripening and (e) particle-particle fusion induced by excess of ultrasonic energy.

To investigate the role of ultrasonic energy on size, a hypothesis is proposed based on our observations and experimental data as shown in Figure 5. Recent studies on the growth mechanism of GNPs have shown that wire-like gold nanoclusters exist on the formation process by extensive nanowire network [24,29,30]. In particular, for a low HAuCl_4_/C_6_H_5_Na_3_O_7_ ratio below 1:3.5, the tendency is towards an extremely fast reaction rate, and consequently, the aggregation of primary particles to the wire-shaped nanoclusters occurs [24]. This aggregation by the nanowire network lasts longer as the concentration of sodium citrate decreases. It can be assumed that ultrasonic energy can physically break the chain-like structure of the gold nanowires. As shown in Figure 6, ultrasonic energy prevented or minimised the randomly self-assembled wire-shaped formation and extensive network of nanowires. This physical effect can expedite sphere-shaped GNPs with Oswald ripening or intra-particle ripening [24]; hence, well-dispersed and monodispersed GNPs can be induced. Moreover, finally formed GNPs can be further dispersed by ultrasonic energy. However, the excess of ultrasonic energy can lead to particle-particle fusion [31] and this can cause a polydispersed state. Thus, suitable ultrasonic energy is the primary parameter to determine particle size and its distribution, as well as the morphology of the nanoparticles. In other words, there is an appropriate and optimal sonication time which contributes to the tunable size of GNPs.

## Conclusions

In summary, highly monodispersed spherical GNPs were produced by the sodium citrate reduction method using a conventional ultrasonic bath without an additional heater or magnetic stirrer. It was found that the sonication energy has a significant effect on the particle size and morphology of GNPs for a fixed ultrasonic power and frequency. Thus, this study shows the importance of ultrasonic energy in the ultrasonic-induced production of water-soluble GNPs of tunable sizes (20 to 50 nm) by citrate reduction. A hypothetical scheme for understanding the role of ultrasonic energy on the size of water-soluble GNPs was discussed. The single-step method using a conventional ultrasonic bath developed in this study offers new opportunities to synthesise aqueous suspensions of monodispersed spherical GNPs without a magnetic stirrer.

The use of ultrasonication without any additional heating and stirring devices is both technologically and scientifically important. Since our results successfully demonstrated that ultrasonication alone is very effective in the synthesis of spherical GNPs, we have developed a simplified method to produce spherical GNPs. Furthermore, because this simplified method involves fewer steps compared to the procedure used by Chen and Wen [26], it is a highly reproducible method for making spherical GNPs of consistent quality. It can, hence, be expected to produce a large volume of consistent quality spherical-shaped GNPs.

Similar experiments using a conventional ultrasonic bath should be performed for other variables, such as the reduction temperature and quantity of sodium citrate.

## Abbreviations

GNPs, Gold nanoparticles; TEM, Transmission electron microscopy; UV, Ultraviolet.

## Competing interests

The authors declare that they have no competing interests.

## Authors’ contributions

JHL proposed the idea, performed the experiments, suggested the scheme and drafted the manuscript. SUSC guided the idea and the experiments, checked the scheme and figures, and finalised the manuscript. SPJ guided the idea and the experiments, checked the figures and revised the manuscript. SYL performed the experiments. All authors read and approved the final manuscript.
